# The Sedative Treatment of Insanity

**Published:** 1849-04-01

**Authors:** 


### THE SEDATIVE TREATMENT OF INSANITY.

In the first number of our Journal, we brought under the notice of the profession the recently published views of Dr. E. J. Seymour on the treatment of certain forms of insanity, by the persevering administration of sedatives. We considered Dr. Seymour's observations on this subject founded on sound physiology, and supported by practical experience. Having witnessed the most extraordinary results from the long continued exhibition of the acetate of morphia, as prescribed by Dr. Seymour, we felt it our duty to direct the attention of those having the care of the insane to this distinguished physician's work. Since the publication of the article in question, we have received from private practitioners, and gentlemen associated with our public institutions, a large body of evidence in support of the treatment recommended by Dr. Seymour. We have before us a paper written by Dr. Frederick Engelken, superintendent of the Asylum for the Insane at Oberneuland, near Bremen, on the exhibition of opiates in various forms of mental derangement. The views of these physicians are strongly corroborative of those entertained by Dr. Seymour. Dr. Engelken would find the acetate of morphia more satisfactory and curative in its effects than any other form of sedative.

The writer, after expressing his unqualified disapproval of the empirical method that had been advocated at some of the scientific meetings of Germany, regarding the exhibition of opium in large doses, gives his opinion that this medicine is, undoubtedly, one of the most admirable remedial agents in psychical diseases. "*During a period of eighteen years*," says Dr. Engelken, "*in which I have employed opium in a large number of cases, both in private practice and in my own establishment, I have seen its administration attended by the most surprising results, and in this respect my opinion of its efficacy is confirmed by the* THIRTY YEARS *experience of my father and predecessor*. Opium is generally favourable to excitable nervous constitutions, and appears to be specially indicated where there is a greater or less degree of erethism of the nerves, and where, consequently, the affection is not of a dynamic nature, but is psychically manifested by a preponderance of exaltation. It should be given in doses of from one to two grains, and gradually increased. In most cases, the object aimed at is attained by giving from three to four grains morning and evening, and it is not often found requisite to increase the doses. The first sleep induced by this exhibition occasionally constitutes a special kind of crisis, as in delirium tremens. An amendment is immediately discoverable, which progresses from day to day. It is worthy of notice, that the excitement which is usually induced by the remedy in question soon wholly disappears, and is succeeded by uninterrupted calm on its continued administration. Where there is only a regular and inconsiderable increase of vascular activity, I have been accustomed to combine *acetum digitalis* with the opium, and to increase the doses until nausea, and even vomiting, was induced, the result of which was, on every occasion, a decided derivation from the brain owing to antagonism."

"Opium exercises a special, or I might almost say specific, power in a condition of disease which I have designated as *melancholia hypochondria*, and which has been admirably described by Dr. G. R. Fleming as *præcordial distress*. I fully concur in all that he has said on the primary and secondary origin of this affection; but I

think, with Professor Schroeder van der Kolk, that the plexus of the sympathetic nerve in the abdomen must also be included, as the seat of the affection. The first stage of præcordial distress is frequently observed in patients under treatment, and is not of unusual occurrence in general practice. It most frequently arises in consequence of violent mental agitation, as sorrow, vexation, annoyance, terror, fear, &c. &c. If the affection be only of recent date, relief may generally be afforded in a very short time; and even in cases of this class, of longer continuance, a successful cure may be effected by the continued exhibition of opium. The dose should be from one to two grains; and if this small quantity does not afford any aid in the treatment, a larger dose will seldom prove of more avail.

"The circumstance that opium generally affords such valuable aid in this præcordial distress, and in *melancholia hypochondria*, may be regarded as a proof of the general nervous nature of the affections. I am confident that, if opium were more frequently given in general practice in this form of disease, the physician would often be enabled, not only to afford immediate relief to the suffering of his patients, but also, in many cases, to prevent præcordial distress from leading to increased melancholy, suicide, or mania. I would here briefly remark, that magnetic electricity affords much benefit in præcordial distress.

"I have long been firmly convinced that opium, when given in small doses, cannot be reckoned amongst narcotics, either in its primary or secondary action, for it evidently increases the activity of the nervous system.

"Schultz Schulzenstein, in his new and admirable Pharmacology, places opium in the same category with wine and ether. It is only when given in larger doses, he writes, that it tends to exert a fatal action. Participating in this view, I have given opium for many years in cholera, with the best possible result; and during the last year I had four cases of this disease in children of poor country people, in three of whom there was decided weakness of mind, and even imbecility. This complication easily supervened, when the patient was not immediately subject to a proper course of treatment.

"The first case was that of a girl aged fourteen. The affection had continued for ten weeks, and the patient had been treated by three different physicians, the last of whom told the parents that, as the medicine prescribed (*aq. laurocerasi*) had produced no effect, the case was incurable. When the girl came under my care, she looked extremely pale and ill; her body was much emaciated, and she could neither sit nor walk, and was obliged to continue lying down: she could not use her hands, and was obliged to be fed, being unable to turn round without assistance. She seemed able to understand the questions put to her, but could not make a suitable reply, and continued to chatter on nonsensically: she was irritable, captious, easily excited to anger, and excessively silly in her manner, gestures, and behaviour. I ordered *op. pur.*, ½ gr.; *rad. valerian*, 8 gr.; *flor. zinci*, 2 grs., to be taken morning and evening. At the end of a week I was informed that a slight amendment had manifested itself; the opium was increased by ¼ of a grain *pro dosi.* In three weeks there was a perceptible amendment, and the opium was again increased ¼ of a grain. At the termination of five weeks, the child had in fact recovered her power of walking and speaking. Doses of 1 grain of opium were continued, and in eight weeks the patient had essentially recovered, and the medicine was stopped. She is now strong and healthy.

"The second case was that of a child aged 10 years. She had had violent toothache, and on an attempt being made to extract the aching tooth, the irritation had attacked the spinal chord, giving rise to chorea. The brain participated so decidedly in the affection, that the child appeared to be quite deranged. A perfect cure was effected in the course of three weeks by opium, given in doses of from ¼ to ¾ of a grain.

"The third case was that of a boy aged 10½ years. A certain degree of feebleness had manifested itself in this patient, showing itself in a slight degree of mental derangement, combined with a peculiar and continuous fretfulness and whining, without shedding tears, and which it was impossible to arrest. A cure was effected in this case at the end of five weeks, and the boy is now perfectly well.

"I could add other cases to these from my earlier experience, but these will be sufficient to draw attention to an admirable means of cure in this form of disease.

"I have employed this medicine, with the most anxious caution, in cases of persons in health as well as in those diseased, and I can truly say that I remember no case, either in insanity or chorea, with or without mental derangement, where it has exerted any directly injurious effect. It is most efficacious in recent, or in any not very chronic cases. I consider it, in general, as extremely dangerous to begin at once with large doses of three grains, and to increase these rapidly, although *occasionally* a cure might be more speedily effected by such a course. Should, however, apoplexy follow on such a mode of treatment as might accidentally happen, it cannot be determined with certainty that this was not the result of the opium. It is only in cases of decided *hyperæsthesis psychica et nervosa*, where the vascular system is but slightly implicated, that larger doses are indicated at the commencement of the treatment."

---

### ON PARTIAL INSANITY.

